# Supplementary material for: A multimodal approach of microglial CSF1R inhibition and GENUS provides therapeutic effects in Alzheimer’s disease mice
Source: bioRxiv. 2025 Jul 16:2025.04.27.648471. Preprint. [Version 2] doi: 10.1101/2025.04.27.648471 (PMC12338498; doi:10.1101/2025.04.27.648471)
Supplement: Supplement 2 [file NIHPP2025.04.27.648471v2-supplement-2.pdf]

bioRxiv preprint doi: https://doi.org/10.1101/2025.04.27.648471; this version posted July 16, 2025. The copyright holder for this preprint (which was not certified by peer review) is the author/funder, who has granted bioRxiv a license to display the preprint in perpetuity. It is made available under aCC-BY 4.0 International license.

SUPPLEMENTARY INFORMATION

A multimodal approach of microglial CSF1R inhibition and GENUS provides therapeutic effects in Alzheimer's disease mice

Chinnakkaruppan Adaikkan[1,2,5,6], Md Rezaul Islam[1,2,6], P. Lorenzo Bozzelli[1,2,6], Matt Sears[1], Cameron Parro[1], Ping-Chieh Pao[1,2], Na Sun[3,4], TaeHyun Kim[1,2], Karim Abdelaal[1,2], Mia Sedgwick[1], Manolis Kellis[3,4], Li-Huei Tsai[1,2,4,*]

Affiliations
[1]Picower Institute for Learning and Memory, Massachusetts Institute of Technology, Cambridge, MA, USA.
[2]Department of Brain and Cognitive Sciences, Massachusetts Institute of Technology, Cambridge, MA, USA.
[3]MIT Computer Science and Artificial Intelligence Laboratory, Cambridge, MA, USA.
[4]Broad Institute of MIT and Harvard, Cambridge, MA, USA.
[5]Present Address: Centre for Brain Research (CBR), Indian Institute of Science, Bangalore 560012, India
[6]Contributed equally: Chinnakkaruppan Adaikkan, Md Rezaul Islam, P. Lorenzo Bozzelli

Correspondence*
Li-Huei Tsai, PhD
Electronic address: lhtsai@mit.edu

bioRxiv preprint doi: https://doi.org/10.1101/2025.04.27.648471; this version posted July 16, 2025. The copyright holder for this preprint (which was not certified by peer review) is the author/funder, who has granted bioRxiv a license to display the preprint in perpetuity. It is made available under aCC-BY 4.0 International license.

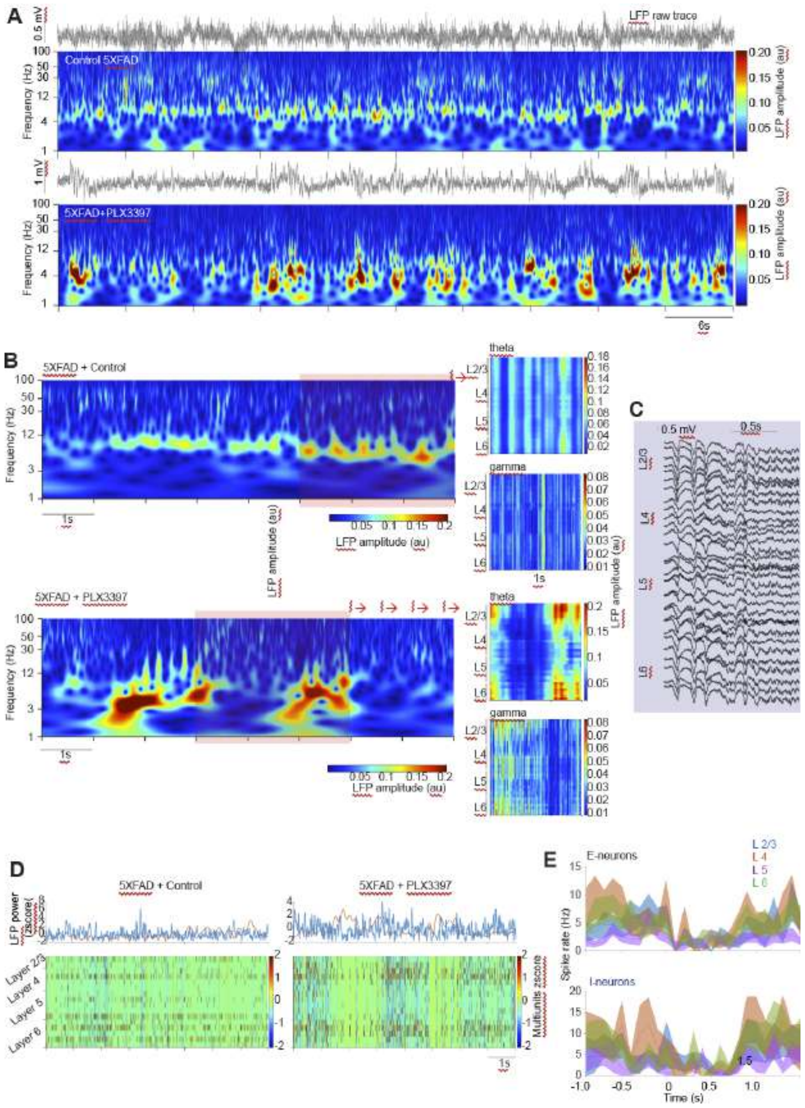

Figure S1. CSF1R-sensitive microglia elimination impacts neural synchrony in 5xFAD mice.

(A) Plots show unprocessed raw LFP traces and the corresponding time-resolved power spectra from 5xFAD without or with PLX3397 administration.

(B) Power spectrogram from layer 4 LFP (left), and LFP theta or gamma power spectra organized according to cortical depth from control and PLX3397-treated 5xFAD mice. L2/3, L4, L5, & L6 indicates cortical layers 2/3, 4, 5, & 6, respectively.

(C) Unprocessed raw LFP traces in PLX3397 5xFAD mice. L2/3, L4, L5, & L6 indicates cortical layers 2/3, 4, 5, & 6, respectively. PLX3397

(D) Top: plots show mean theta (3- 12 Hz) and gamma (30-50 Hz) power in 5xFAD without or with Plx3397PLX3397 administration. Multiunit activity is shown at the bottom.

(E) Line plots show the mean (± s.e.m) spike rate of E-neurons (top) and I-neurons (bottom) pre-, during-, and post-high theta activity from L2/3, L4, L5, and L6. Time zero represents high theta activity onset.

bioRxiv preprint doi: https://doi.org/10.1101/2025.04.27.648471; this version posted July 16, 2025. The copyright holder for this preprint (which was not certified by peer review) is the author/funder, who has granted bioRxiv a license to display the preprint in perpetuity. It is made available under aCC-BY 4.0 International license.

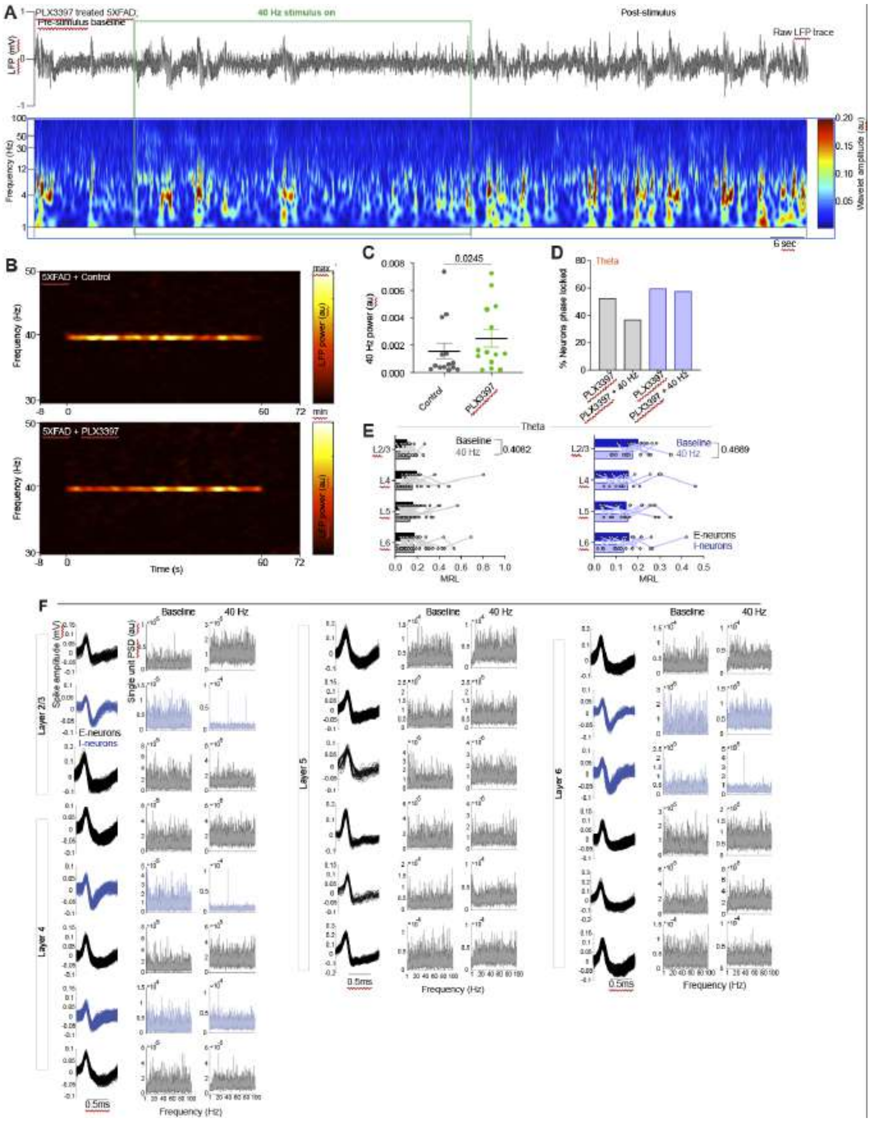

Figure S2. Sensory evoked gamma oscillations in PLX3397 treated 5xFAD mice.

(A) LFP trace (top) during pre-stimulation in PLX3397-treated 5xFAD mice — the corresponding wavelet LFP spectrogram before, during, and after acute 40 Hz stimulation. Note the reduction in theta activity during the 40 Hz entrainment.

(B) LFP power spectrum in 5xFAD mice with or without PLX3397 administration for 50 days. PLX3397 administered 5xFAD mice exhibited clear 40 Hz entrainment during acute 60sec stimulation.

(C) The summary graph shows the mean power of 40 Hz entrainment in control and PLX3397 administered 5xFAD mice.

(D) Plot shows the percentage of total neurons phase-locked to theta oscillations based on circular Rayleigh statistics.

(E) Plots show the strength of phase locking between neuronal spiking and LFP theta. No significant effect was observed in E-neurons ($F(1, 97) = 0.6899$, $P = 0.4082$) and I-neurons (ANOVA, $F(1, 43) = 0.4873$, $P = 0.4889$) between baseline and 40 Hz entrainment.

(F)Twenty simultaneously recorded single units are organized according to cortical layers in PLX3397+GENUS-treated mice. Spike waveforms of isolated units and the power spectral density of units are shown.

bioRxiv preprint doi: https://doi.org/10.1101/2025.04.27.648471; this version posted July 16, 2025. The copyright holder for this preprint (which was not certified by peer review) is the author/funder, who has granted bioRxiv a license to display the preprint in perpetuity. It is made available under aCC-BY 4.0 International license.

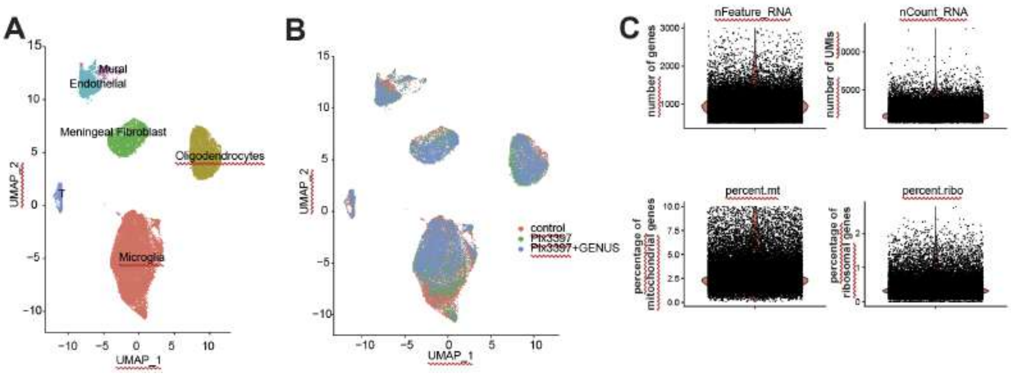

Figure S3. Single-cell transcriptomics profiling from the visual cortices of 5xFAD mice.

(A) UMAP plot displaying cell clusters corresponding to microglia, oligodendrocytes, meningeal fibroblasts, endothelial cells, mural cells, and T cells.

(B) UMAP plot demonstrating that the cell clusters are well integrated across different treatment conditions.

(C) Violin plots showing the distribution of quality control metrics, including the number of detected genes, UMIs, and the percentage of mitochondrial and ribosomal gene expression.

bioRxiv preprint doi: https://doi.org/10.1101/2025.04.27.648471; this version posted July 16, 2025. The copyright holder for this preprint (which was not certified by peer review) is the author/funder, who has granted bioRxiv a license to display the preprint in perpetuity. It is made available under aCC-BY 4.0 International license.

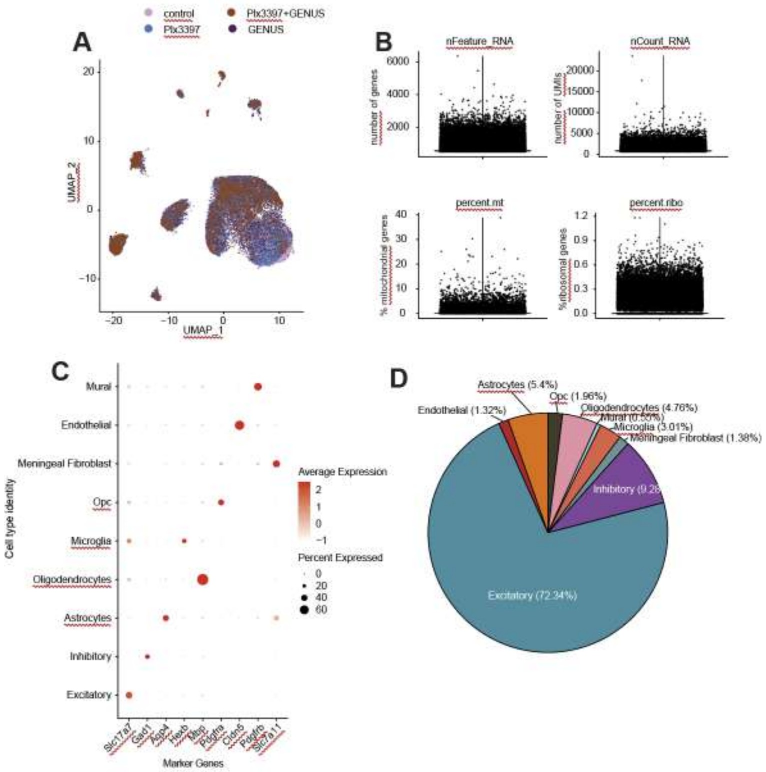

Figure S4. Single-nuclei transcriptomics profiling from the visual cortices of 5xFAD mice.
(A) UMAP plot demonstrating well-integrated cell clusters across different treatment conditions.
(B) Violin plots depicting the distribution of quality control metrics, including the number of detected genes, UMIs, and the percentage of mitochondrial and ribosomal gene expression.
(C) Dot plots illustrating the expression of cell-type-specific marker genes within their respective cell types.
(D) Pie chart showing the proportional distribution of identified cell types analyzed in the dataset.

bioRxiv preprint doi: https://doi.org/10.1101/2025.04.27.648471; this version posted July 16, 2025. The copyright holder for this preprint (which was not certified by peer review) is the author/funder, who has granted bioRxiv a license to display the preprint in perpetuity. It is made available under aCC-BY 4.0 International license.

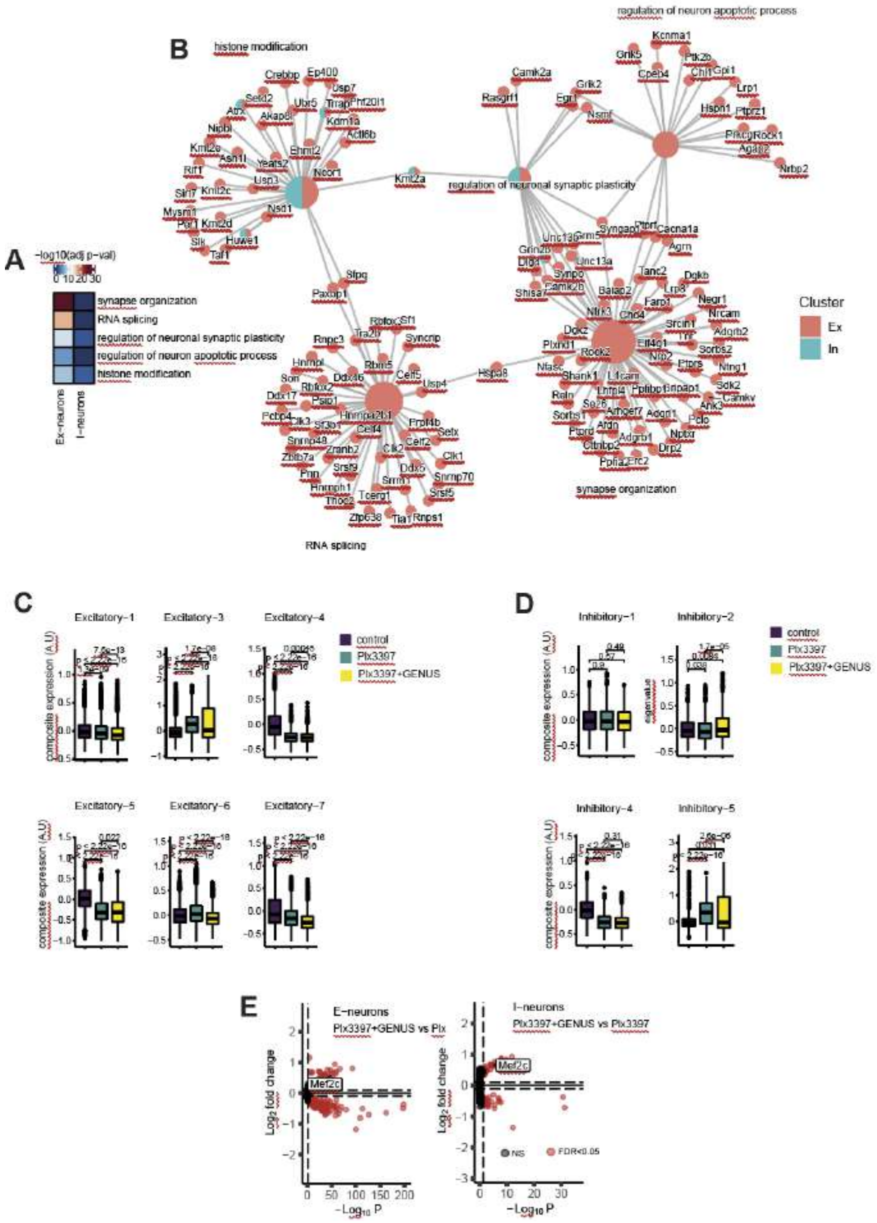

Figure S5. Administration of chronic gamma entrainment enhances Mef2c in both E- and I-neurons of PLX3397 treated 5xFAD mice.

(A, B) Gene ontology (GO) analysis for the downregulated genes comparing PLX3397+GENUS and PLX3397 alone in E- and I-neurons. (A) Top GO terms represented in E- and I-neurons for the downregulated genes. (B) Gene network with nodes and edges highlighting the genes involved in the corresponding GO term. Larger nodes represent the gene ontology term while the smaller nodes highlight the genes. Red and green colors represent the observations in the E- and I-neurons respectively.

(C, D) Expression of gene modules for (C) excitatory and (D) inhibitory neurons among experimental groups. The midline of the Boxplots indicates the median, while the lower and upper lines represent the 25th and 75th percentiles, respectively. The whiskers represent the smallest and largest values in the 1.5× interquartile range. Kruskal-Wallis test.

(E) Volcano plots highlight Mef2c as significantly upregulated in both E- and I-neurons.

bioRxiv preprint doi: https://doi.org/10.1101/2025.04.27.648471; this version posted July 16, 2025. The copyright holder for this preprint (which was not certified by peer review) is the author/funder, who has granted bioRxiv a license to display the preprint in perpetuity. It is made available under aCC-BY 4.0 International license.

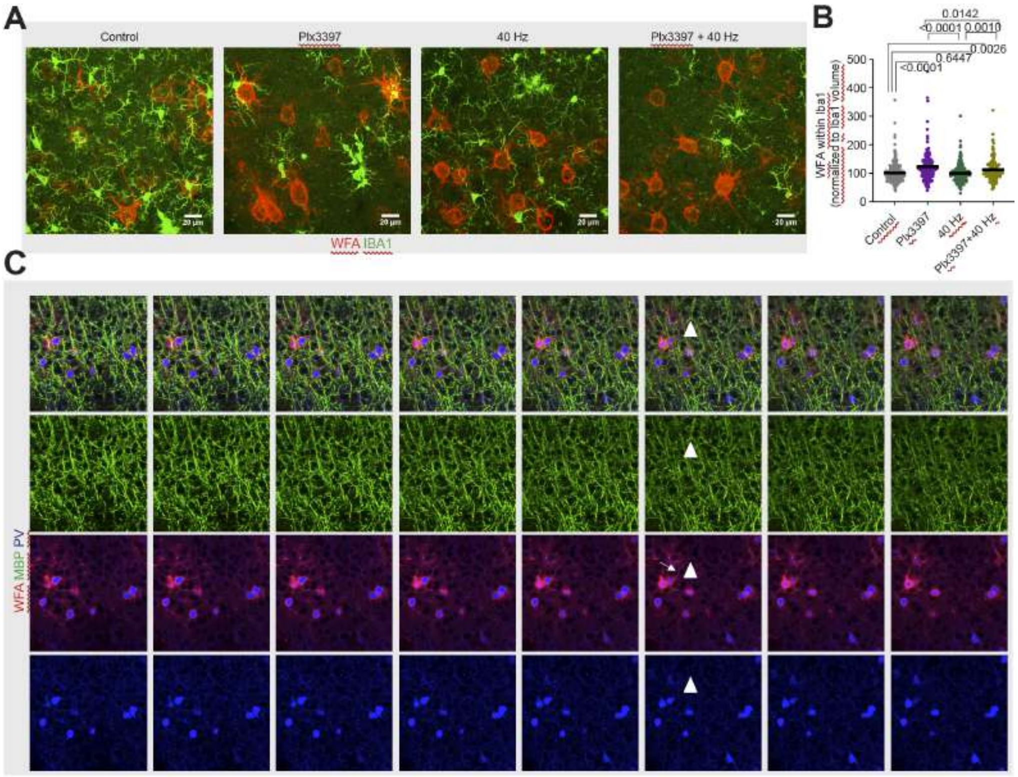

Figure S6. Chronic administration of PLX3397 and GENUS perineuronal net.
(A) Representative confocal images show WFA and IBA1 (scale bar = 20 μm).
(B) Plot shows the WFA signal within IBA1 (ANOVA F= 17.96, p < 0.0001).
(C) Representative serial single-plane confocal images show WFA, MBP, and PV. Note that MBP signals around the axonal process of PV interneurons are evident immediately after WFA but not within WFA. This suggests a multifaceted regulation of the PV axon through myelination and PNN.

bioRxiv preprint doi: https://doi.org/10.1101/2025.04.27.648471; this version posted July 16, 2025. The copyright holder for this preprint (which was not certified by peer review) is the author/funder, who has granted bioRxiv a license to display the preprint in perpetuity. It is made available under aCC-BY 4.0 International license.

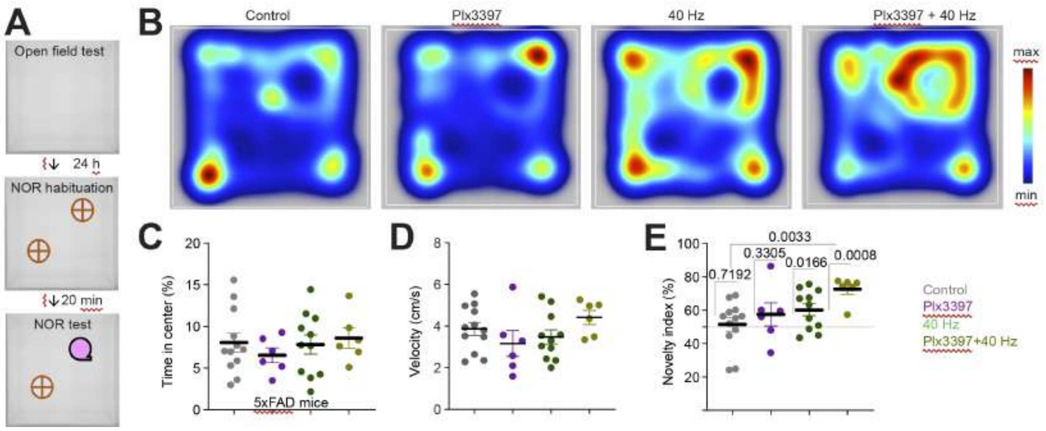

Figure S7. Chronic administration of PLX3397 and GENUS improves novel object recognition memory in 5xFAD mice.

(A) Schematic of OF and NOR test.

(B) Mice occupancy heatmaps during the NOR test in 5xFAD mice.

(C) Time spent in the center during OF (c, ANOVA, F (3, 31) = 0.3847, P = 0.7647) did not differ between groups.

(D) Plot shows the velocity of mice during a novel object recognition memory test in 5xFAD mice (ANOVA F (3, 31) = 1.437, P = 0.2508.

(E) Novelty index during NOR test was higher in PLX3397+GENUS-treated 5xFAD mice (ANOVA, F (3, 31) = 3.456, P = 0.0282).
